# Hebbian Optocontrol of Cross-Modal Disruptive Reading in Increasing Acoustic Noise in an Adult with Developmental Coordination Disorder: A Case Report

**DOI:** 10.3390/brainsci14121208

**Published:** 2024-11-29

**Authors:** Albert Le Floch, Guy Ropars

**Affiliations:** Unité de Formation et de Recherche Sciences et Propriétés de la Matière, University of Rennes, Campus de Beaulieu, Building 7, Laboratory P938, 35042 Rennes Cedex, France; albert.lefloch@laposte.net

**Keywords:** acoustic noises in reading disorders, acoustic and visual noise interactions, optocontrol

## Abstract

Acoustic noise is known to perturb reading for good readers, including children and adults. This external acoustic noise interfering at the multimodal areas in the brain causes difficulties reducing reading and writing performances. Moreover, it is known that people with developmental coordination disorder (DCD) and dyslexia have reading deficits even in the absence of acoustic noise. The goal of this study is to investigate the effects of additional acoustic noise on an adult with DCD and dyslexia. Indeed, as vision is the main source of information for the brain during reading, a noisy internal visual crowding has been observed in many cases of readers with dyslexia, as additional mirror or duplicated images of words are perceived by these observers, simultaneously with the primary images. Here, we show that when the noisy internal visual crowding and an increasing external acoustic noise are superimposed, a reading disruptive threshold at about 50 to 60 dBa of noise is reached, depending on the type of acoustic noise for a young adult with DCD and dyslexia but not for a control. More interestingly, we report that this disruptive noise threshold can be controlled by Hebbian mechanisms linked to a pulse-modulated lighting that erases the confusing internal crowding images. An improvement of 12 dBa in the disruptive threshold is then observed with two types of acoustic noises, showing the potential utility of Hebbian optocontrol in managing reading difficulties in adults with DCD and dyslexia.

## 1. Introduction

Many studies have investigated the effects of environmental acoustic noise on school-aged children [1,2,3,4,5,6] and adults [7,8]. In particular, the influence of acoustic noise has been shown to be detrimental on reading performance with some disturbance in reading comprehension [9,10]. Moreover, children with learning disorders are still more impaired by acoustic noise [11,12,13]. Indeed, the cross-modal processing in early visual and auditory cortices is well established [14,15,16], with a multimodal convergence of information from sensory to associative areas in the brain [17,18,19]. In readers without learning disorders, background acoustic noise appears as a single external perturbation at the multimodal brain areas. However, when a lack of asymmetry exists between the Maxwell centroids and the topographies of cones in the two foveas, namely, for dyslexic readers, vision itself can be disturbed as the primary images on the primary cortex are often accompanied by extra mirror or duplicated images [20,21]. In this case, this internal visual crowding behaves like a second noise, which is embarrassing for a person without asymmetry between their two eyes and without ocular dominance, inducing reading impairments. This visual crowding forces the dyslexic reader to make more eye saccades and fixations, which can be recorded using an eye tracker [22]. Many groups have already observed this excess of fixations and saccades in dyslexic observers and suggest these observations as a diagnosis for reading disorders [22,23,24,25,26,27].

In the presence of both an external acoustic background noise and an internal visual crowding converging in the multimodal areas of the brain, one might expect an abrupt disruption in reading with an increased number of fixations when the external acoustic noise is regularly increased. First, we investigate such disruption suffered by a young adult with DCD and dyslexia at a given level of an increasing acoustic noise when reading a text. Second, we use an eye tracker to characterize the disruption at two levels of the external increasing acoustic noise, below and above the observed disruption threshold. Third, we show that the disruption threshold can be pushed back when the visual internal crowding is erased by Hebbian mechanisms at the primary cortex synapses, using liquid crystal glasses providing a pulsed-lighting regime. The results are compared to those obtained by a typical reader in the presence of the same types of acoustic noises. These results introduce enhancing support strategies for individuals with DCD and dyslexia.

## 2. Methods

### 2.1. Subjects

A 21-year-old male student from our university with co-occurring developmental coordination disorder and dyslexia participated in the experiments. This student has several difficulties, in coarse and fine motor skill coordination, in reading and writing, and in the ability to plan. One other male student (also 21 years old), attending the same physics courses in the third year of a bachelor’s degree program at the University of Rennes, participated in the same experiments as a control, being a typical reader. Both are native French speakers and have normal binocular vision. The two participants in this study gave written informed consent. The entire study was conducted according to the principles expressed in the Declaration of Helsinki.

### 2.2. Comparison of the Two Maxwell Centroids with a Foveascope

**Figure 1:**
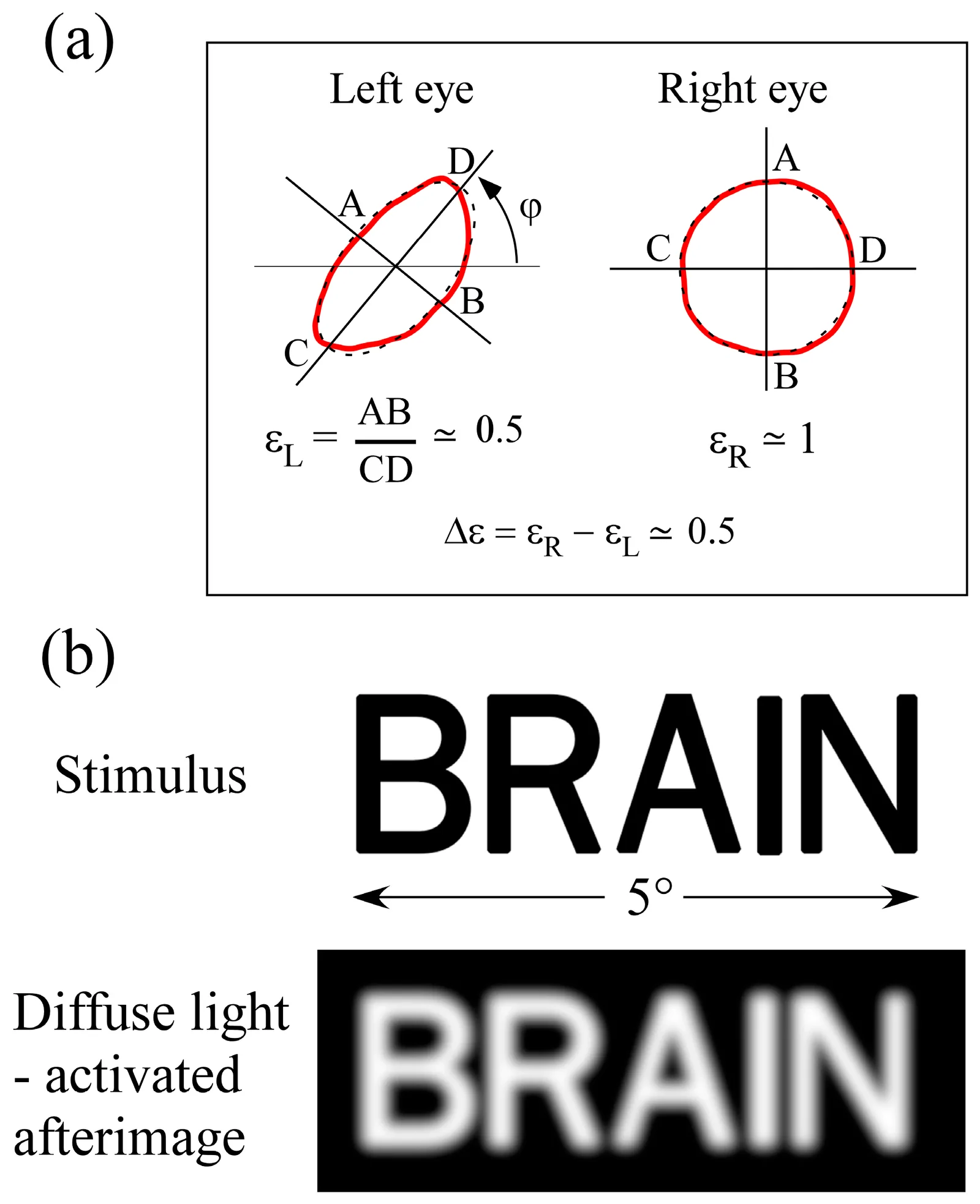
(**a**) Maxwell centroid outlines for a typical reader. The diameters of the real centroids on the fovea are about 100 to 150 μm, but here on the screen of the foveascope, the enlarged outlines are about 3 cm. The circular outline observed for the right eye determines the ocular dominance. The typical asymmetry Δε reaches + 0.5 for the right-eye-dominant observer. (**b**) After fixation on the stimulus—i.e., the word BRAIN—the diffuse light-activated afterimage is observed through closed eyelids. The reconstructed afterimage is perceived by the observer.

**Figure 2:**
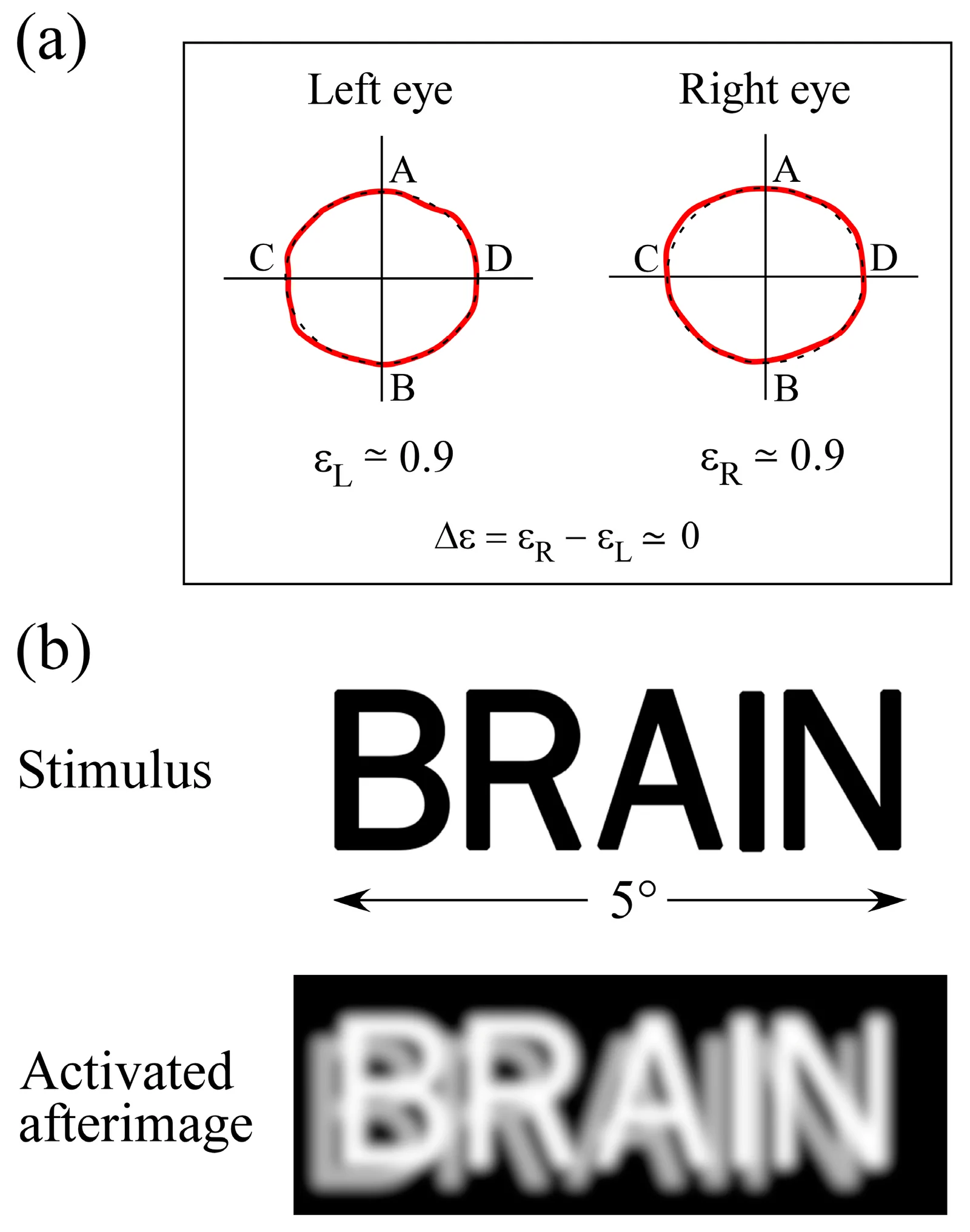
(**a**) Same recordings as in Figure 1a but for an observer with DCD and dyslexia. Here, no asymmetry exists between the two centroids. (**b**) The activated afterimage shows the noisy superposition of both the primary and the duplicated images.

To introduce the noisy visual crowding affecting many observers with dyslexia, we have to record the lack of asymmetry of their Maxwell centroids [20]. The Maxwell centroids are located at the center of the spot earlier observed by Maxwell [28]. They correspond to a small blue-cone-free area with a diameter of about 100 to 150 μm at the center of each fovea. The first direct observations have been performed post-mortem [29,30] after collecting intact retinas and staining the blue cones with specific antibodies. However, observing any asymmetry between the outlines of the Maxwell centroids was out of reach, owing to the splitting and swelling in the retinal tissues. To record and compare the two Maxwell centroids for each observer, we have built a foveascope [20] capable of visualizing and enlarging entoptic images of the blue-cone-free areas in the blue part of the spectrum. To optimize the contrast of the entoptic images, we used a blue–green exchange filter that alternates between the two colors with a periodicity of about 7 s. An image of about 3 cm is then obtained for a distance of 3 m between the observer and the screen. For the control, an asymmetry between the two outlines is observed (Figure 1a). The quasi-circular outline corresponds to the dominant eye, while the elliptical outline—here with an inclination φ of its main axis, which is about 45°—corresponds to the non-dominant eye. The difference in ellipticity between the two outlines $\Delta \varepsilon =\varepsilon_{R}-\varepsilon_{L}$ is typically about $\left|0.5\right|$ . For right-eye-dominant observers, the quasi-circular outline is seen in the right eye with a prevalence of about two for three, while for left-eye-dominant observers, the quasi-circular outline is seen with the left eye with a prevalence of about one for three. The two outlines for the student with DCD and dyslexia are shown in Figure 2a. In contrast, here both outlines are quasi-circular with an ellipticity of 0.9. This observer has a lack of asymmetry and no ocular dominance. His diffuse light-activated negative afterimages have the same luminance. Indeed, after a binocular fixation for about 10 s on a contrasted stimulus, an observer perceives through his closed eyelids, in each eye, an afterimage with its specific luminance. The higher luminance corresponds to the dominant eye for a typical reader, while for a dyslexic reader, the illuminance is the same for the two eyes.

### 2.3. Noisy Internal Crowding

The diffuse light-activated afterimage method provides further information concerning neural connectivity in the brain. After binocular fixation on a contrasted stimulus such as the word BRAIN, for 5 to 10 s under 5000-lux daylight, clear afterimages are perceived. While the observer without DCD or dyslexia only perceives the primary image of the word BRAIN (Figure 1b), the observer with DCD and dyslexia perceives both the primary afterimage but also the duplicated word generally slightly shifted (Figure 2b). Note that in many cases, a dyslexic observer can perceive a mirror image instead of a duplicated image [21]. Obviously, the extra superposed images induce an internal visual crowding that acts as an internal noise for the dyslexic observer, leading to reading difficulties with an excess of visual fixations and longer durations of fixations. These extra superposed images have been shown to be erasable for a dyslexic person via Hebbian mechanisms using pulsed lighting [20]. The pulse frequency is optimized for each person to take into account a small delay of about 10 ms [31] that exists between the apparition of the primary image and the mirror or duplicated image that has to travel through the corpus callosum, which connects the two hemispheres of the brain. For the student with DCD and dyslexia, this optimized frequency corresponds to 82 Hz when his extra duplicated images are erased. The optimal frequency is obtained by continuously varying the frequency in the 60 to 120 Hz range. Note that the use of such relatively high frequency avoids any disturbing visible flickering.

### 2.4. The Two Types of Acoustic Noise Used in the Experiments

A Cirrus Research plc Optimus red sound level meter, model CR-162C, was used to analyze noise. All the functions of the instrument are measured simultaneously across a wide range of 120 dB. This sound level meter has real-time octave band filters that measure noise in 10 different frequency bands. The octave band measurement is taken at the same time as the other measurements and includes the overall level in each band together with a time history of the band across the measurement period. We used Noise Tolls software on a PC to download, analyze, and report the noise measurement information. Two types of acoustic noises were used, i.e., a computer keyboard noise and an ambient noise in a hall. Indeed, the student with DCD and dyslexia drew our attention to his difficulties, particularly in computer classes, because of the environmental noise from the keyboards. The two noises were selected from an internet sound library [32], as these two noises are both common but have clearly different intensity variations versus frequency. During the experiments, the noise intensity—taking into account 28 dBa of background noise in the room—was varied linearly from 30 to 70 dBa for about 20 s, making it possible to detect any disruption when the reader tries to read a text displayed on a computer screen or text written on a sheet of paper.

### 2.5. Eye Tracking Experiment for Observing the Role of Acoustic Noise

The first reading experiment, which detected and characterized the disruption threshold when the keyboard noise was regularly increased, was carried out using a HP Compaq LE 2202X computer screen equipped with an infrared eye tracking system. The eye tracker used was a commercial infrared system (Tobii dynavox PCEye Plus, version 1.3) with a sampling frequency of 60 Hz, which was used with software (Tobii Dynavox Gaze viewer, V.1.2.0.63881) for rending data as images and movies with gaze plots. The eye tracking analysis algorithm provides the total number of fixations. Calibration was carried out using a 9-point routine. The participants were seated about 60 cm from the screen, which is the optimal range for recording, as described in the eye tracker manual. A chin and forehead rest was used to limit head movements. The experiment was carried out in a dark room. A regularly increasing noise was generated by a keyboard noise in a computer room and electronically amplified at levels varying from 30 dBa to 70 dBa. Noise levels were continuously measured using the Cirrus Research sound level meter.

### 2.6. Glasses Used for the Reading Task in Continuous and Pulsed-Light Regimes

In the second experiment, which investigates Hebbian optocontrol of the disruption threshold for silent reading of text written on a sheet of paper in the presence of external acoustic noise, was performed using two pairs of glasses. The two pairs are apparently similar (see below). The first pair equipped with neutral filters works in a standard continuous regime adapted to transmit the same illuminance as the second pair of glasses. This second pair of glasses is equipped with liquid crystals that are electronically controlled to periodically modulate the transmitted light at frequencies varying from 60 to 120 Hz with a duty cycle of 0.2 (see below). Here, the frequency chosen was 82 Hz, corresponding to the optimization for the DCD-with-dyslexia student. This second reading test was performed with a text on a sheet of paper under a daylight intensity of 7000 lux. The acoustic noise perturbation in this second experiment was produced by the same system as in the first experiment. However, here we used two types of noises: a keyboard noise and a hall noise with their corresponding noise fluctuations and spectra measured using the Cirrus apparatus.

## 3. Results

### 3.1. Observation of the Disruption Threshold in the Presence of Increasing Acoustic Noise

**Figure 3:**
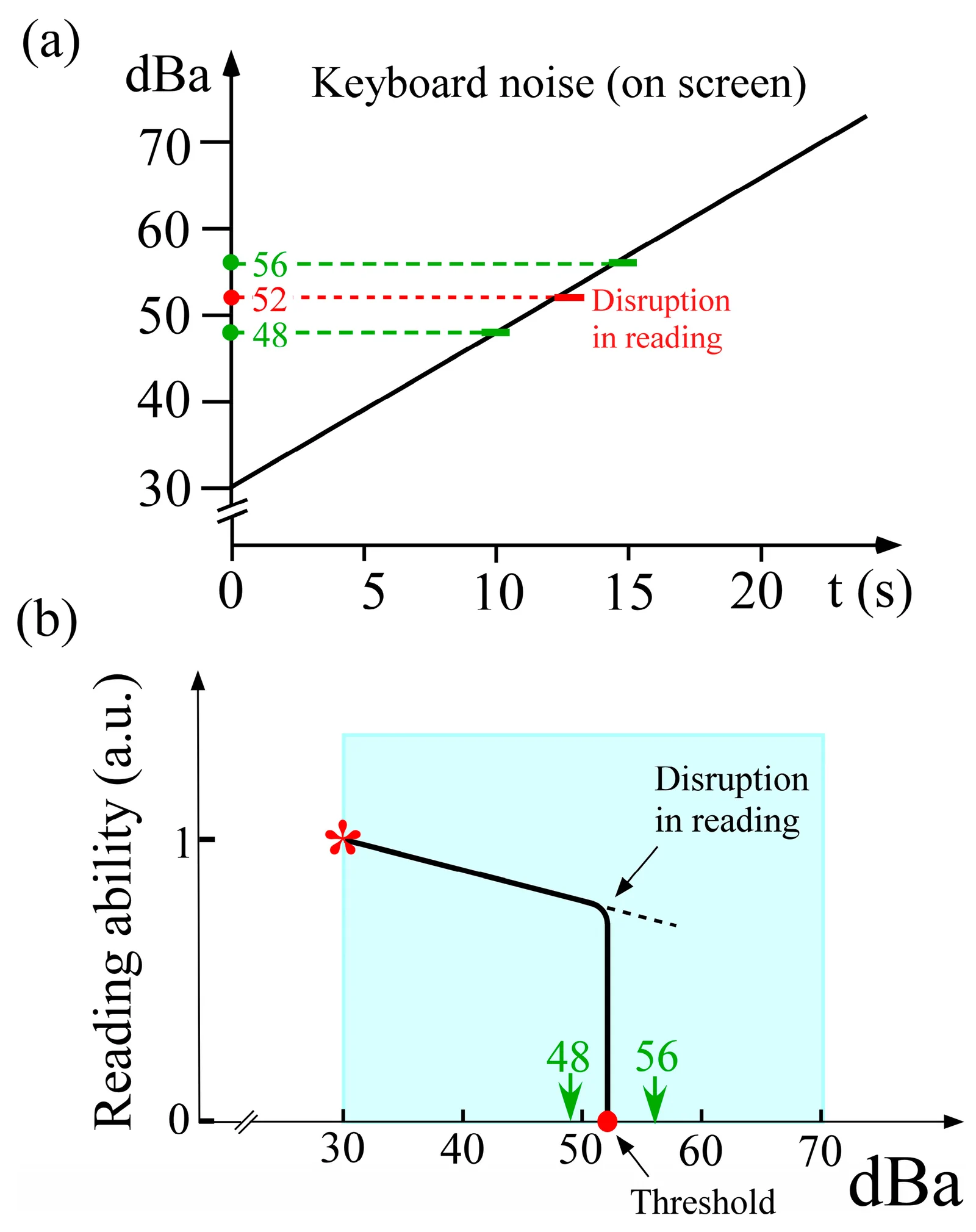
(**a**) Observation of the disruption in reading by the observer with DCD and dyslexia when the acoustic noise was linearly increased versus time. The background level of the noise in the room was 28 dBa. The precision of the noise levels in our experiments is estimated to ±2 dBa. (**b**) Qualitative reading ability of the student when the acoustic noise was varied in the 30–70 dBa range. In this experiment, the student with DCD and dyslexia verbally pointed out when his understanding and comprehension were completely disrupted. The specific instructions were simple: a vocal signal was given to start the silent reading, and the student gave a vocal signal (“stop”) when he reached the disruption threshold.

In the first experiment, the silent reading task consisted of reading text with letters with a size of 3.5 mm at a distance of 60 cm on an HP computer screen. For the reader without DCD and dyslexia, reading remained unperturbed when the keyboard noise was increased in the 30 to 70 dBa range, while the student with DCD and dyslexia rapidly confirmed his difficulties. He experienced an abrupt disruption with complete loss of comprehension when the level of keyboard noise reached 52 dBa (Figure 3a,b).

### 3.2. Characterization of the Disruption Threshold by the Eye Tracker

**Figure 4:**
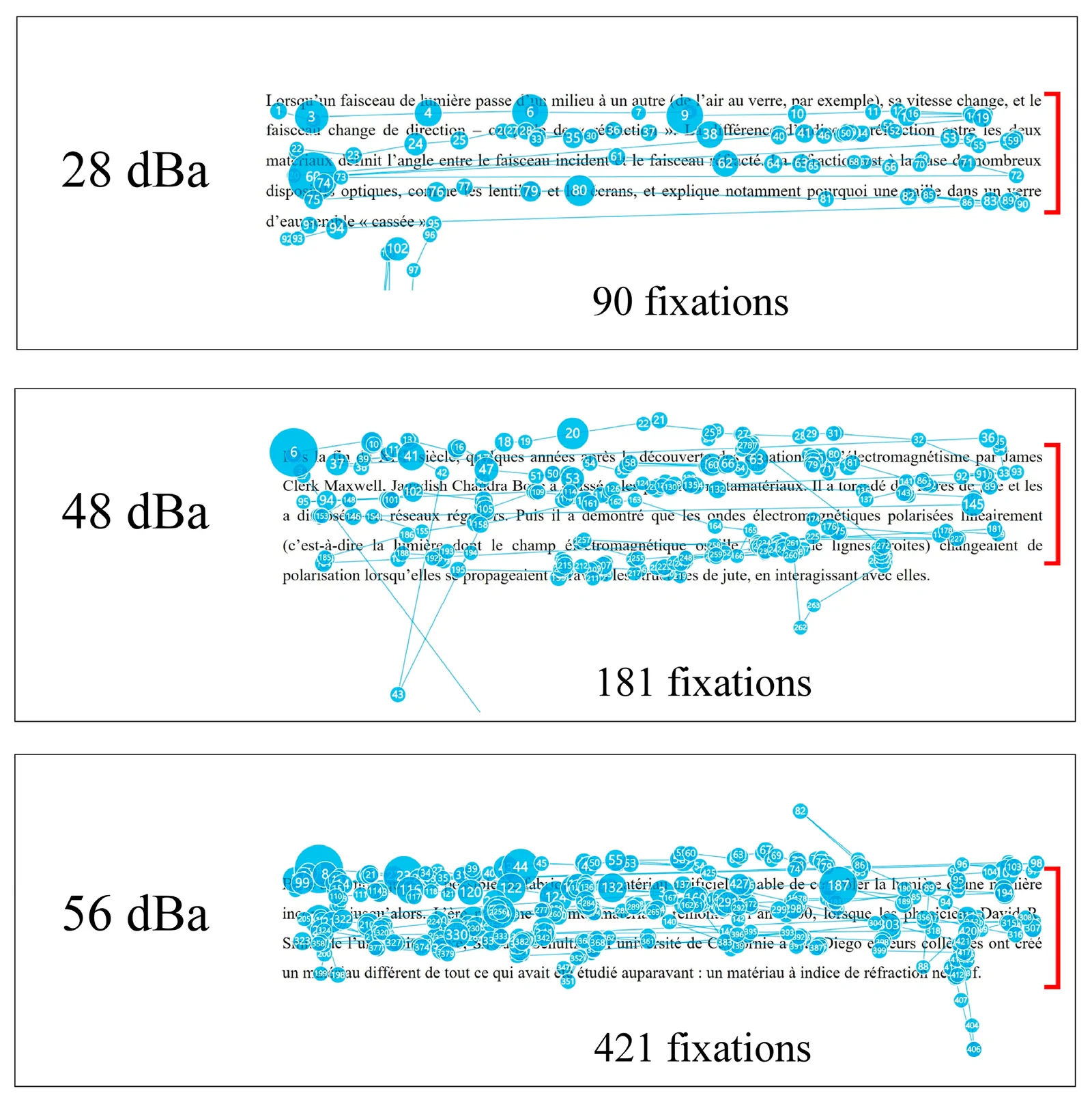
Eye tracking measurements for the student with DCD and dyslexia when reading five lines of text on the HP screen. Without added acoustic noise at 28 dBa corresponding to the background noise in the room, 90 fixations are necessary to read the first four lines. Under the disruption threshold, the fixation number remains at 181, while above the threshold, the 421 fixations illustrate chaotic reading patterns.

**Figure 5:**
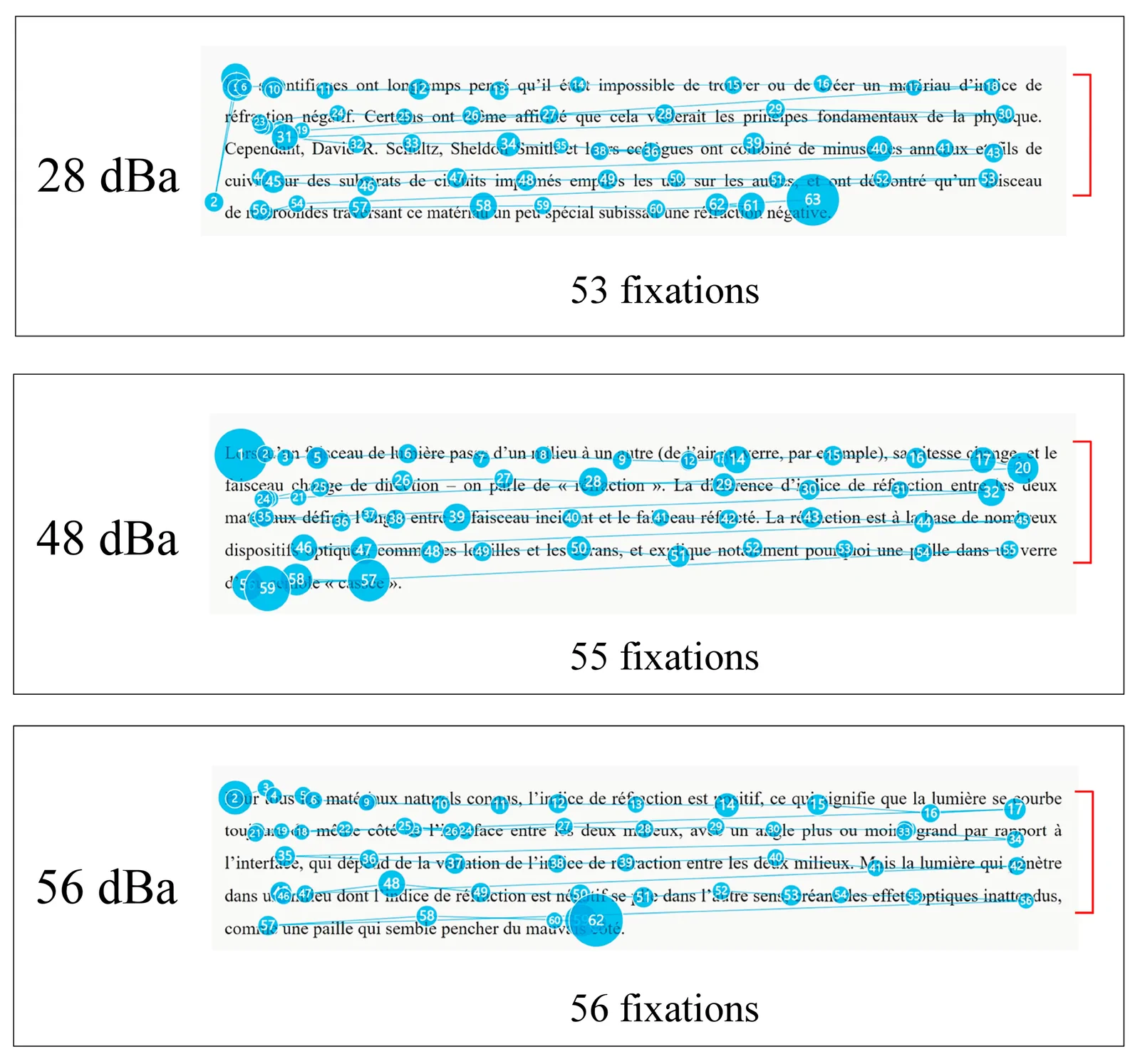
Eye tracking measurements for the typical reader. Without added acoustic noise, 53 fixations are necessary to read the first four lines. At 48 and 56 dBa, only a slight increase in the number of fixations is observed.

**Figure 6:**
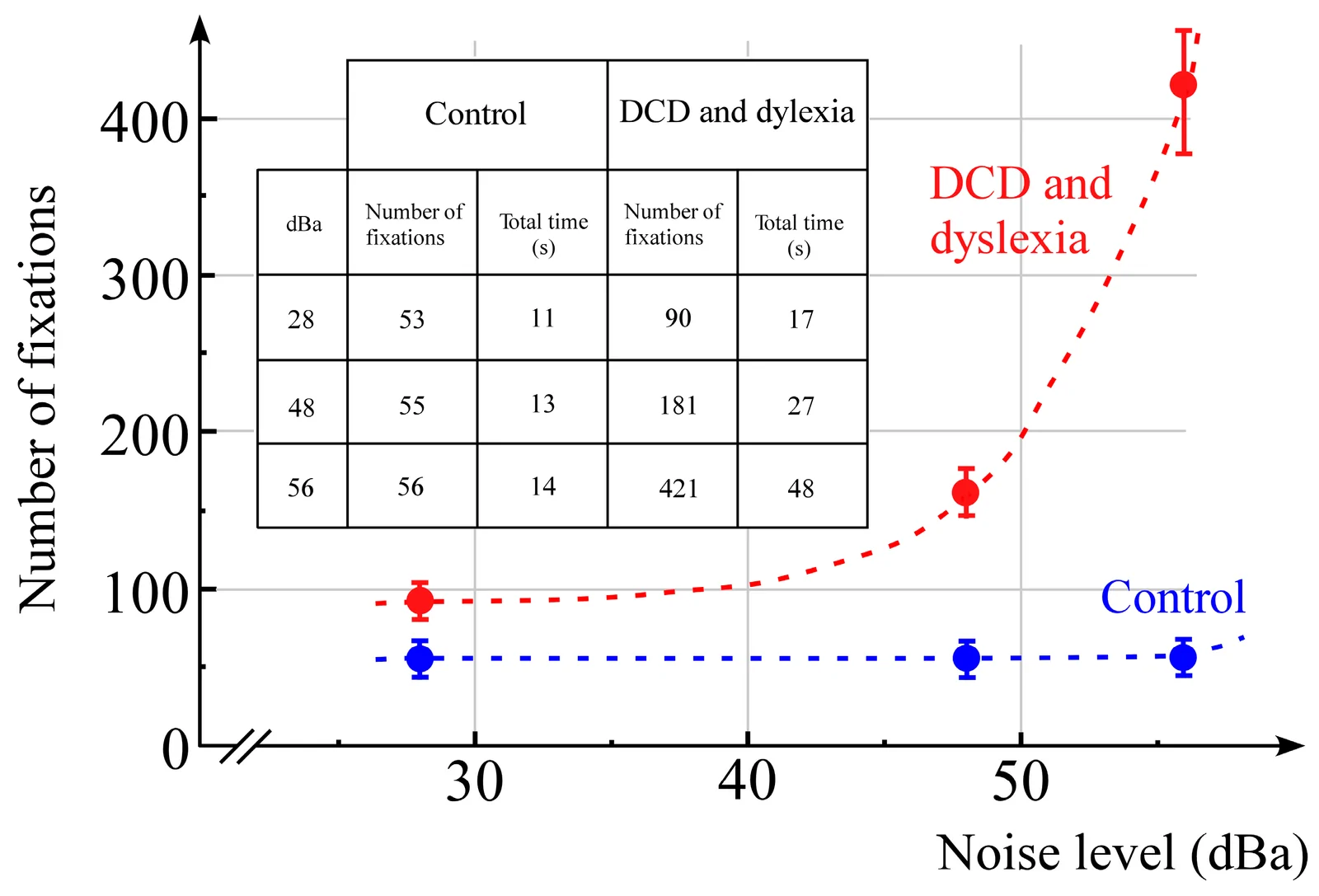
Comparative number of fixations recorded for the DCD and dyslexia observer and for the typical reader in the 30 to 70 dBa range. In the absence of added acoustic noise, the student with DCD and dyslexia still shows a higher number of fixations than the typical reader, and this number sharply increased around 50 dBa. The error bars represent the standard deviations.

In order to characterize this disruption, we have recorded the number of fixations below and above this threshold at 48 dBa and 56 dBa of acoustic noise intensities, respectively. The corresponding results are shown in Figure 4 when the student with DCD and dyslexia read a five-line text. Below the threshold, the number of fixations is 181, while above the threshold, the number of fixations reaches 421, indicating chaotic disorder. The eyes become unable to follow the lines of the text. In contrast, for the typical reader, varying the acoustic noise level from 30 to 70 dBa did not show any disruption (Figure 5). The acoustic noise only slightly increased the number of fixations. The comparison of the evolutions of the number of fixations is shown in Figure 6 for the two students. While the number of fixations is even larger in absence of the added external acoustic noise, the sharp increase around 50 dBa is clear for the student with DCD and dyslexia.

### 3.3. Optocontrol of the Disruption Threshold in the Presence of Increasing Acoustic Noises

In this second experiment, consisting of reading a sheet of paper with text, we use liquid crystal glasses to produce a pulsed-lighting regime. This experiment was carried out under daylight of about 7000 lux illuminating a standard text with letters 1.5 mm in size, corresponding to an angular dimension of about 13 min of angle for each letter.

**Figure 7:**
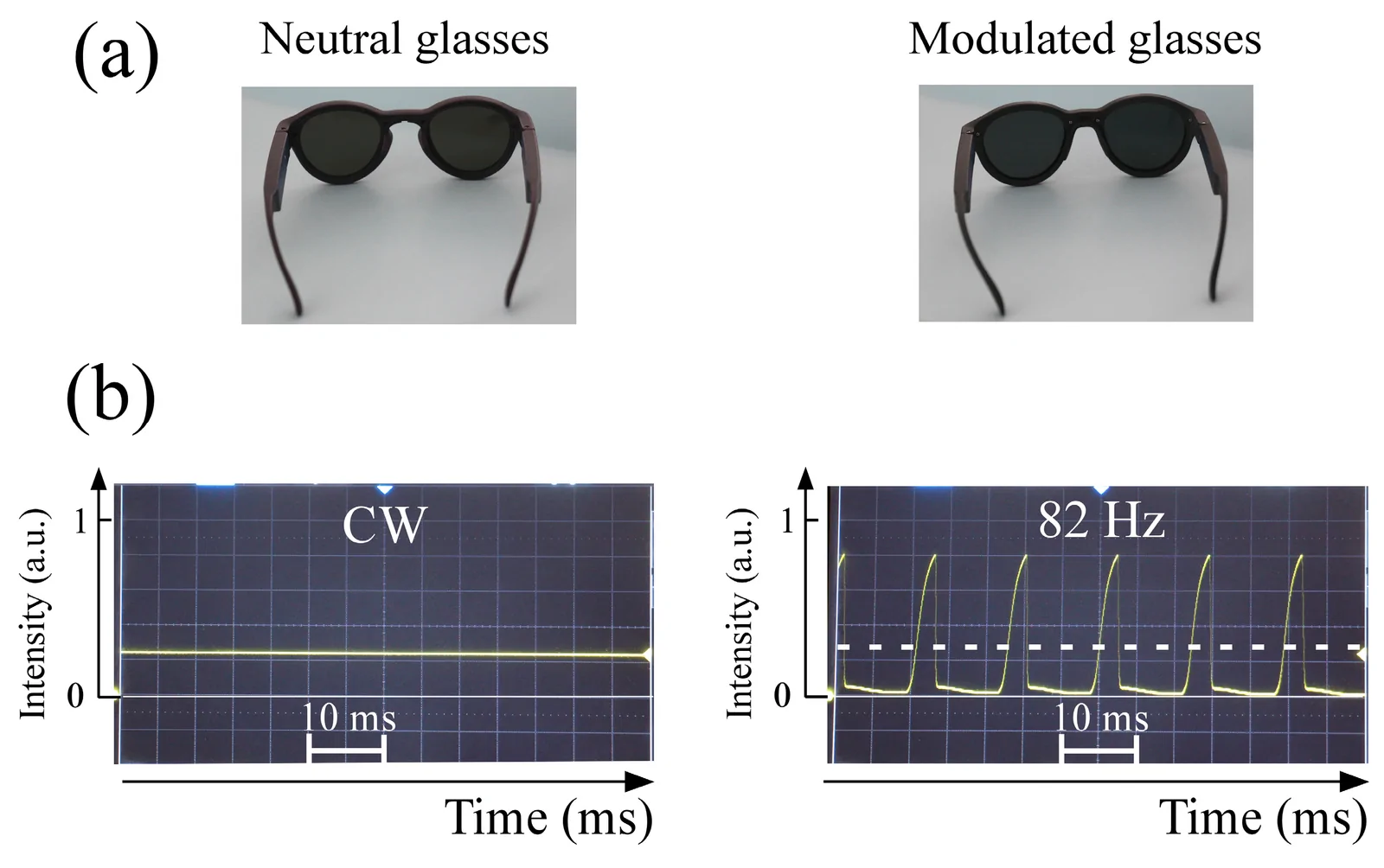
(**a**) Photos of the neutral and modulated glasses showing their similarity. (**b**) The intensity transmitted by the neutral glasses has been adjusted so as to be strictly equal to the mean intensity transmitted through the modulated glasses, shown by the dotted line.

**Figure 8:**
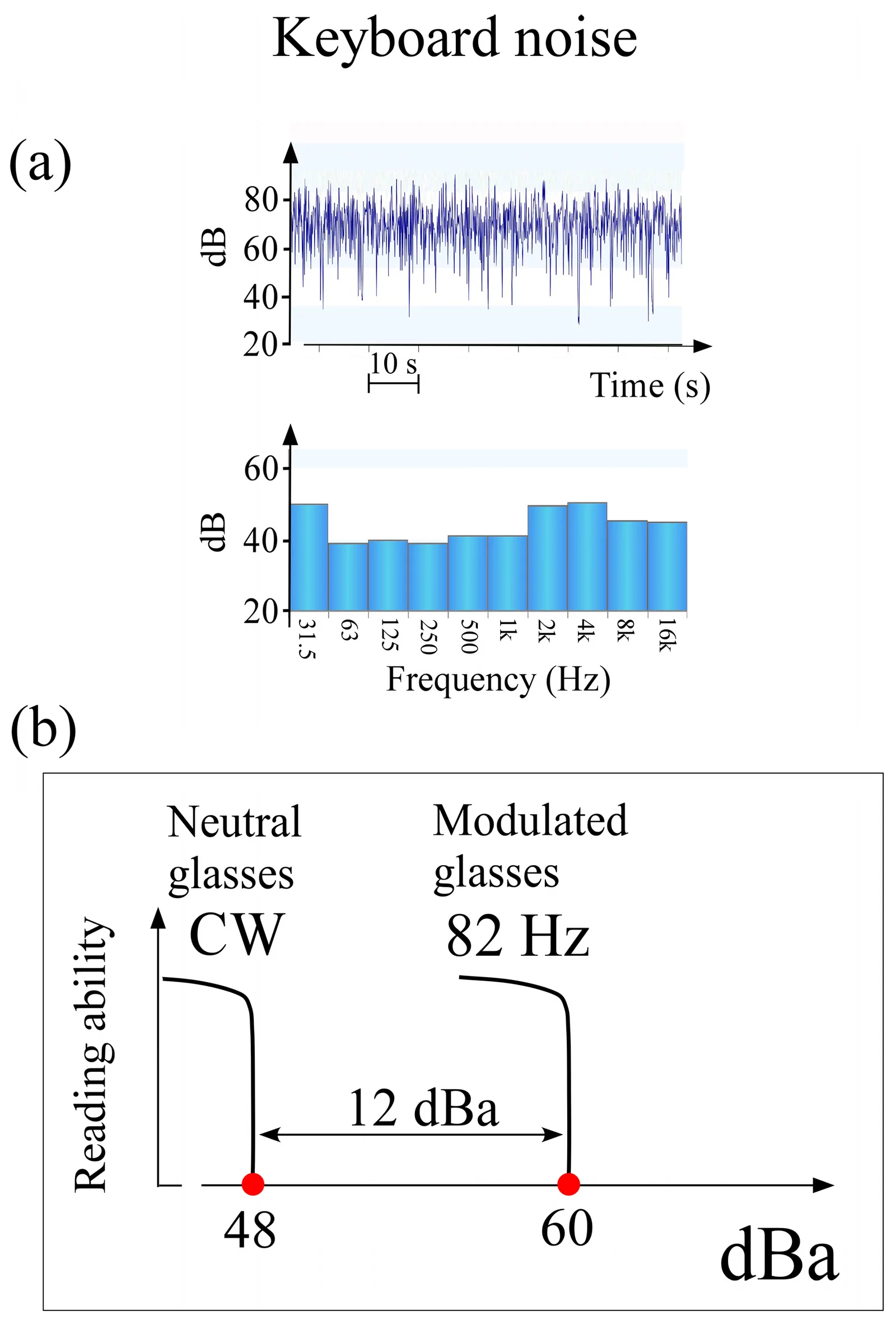
(**a**) Keyboard noise characteristics showing the fluctuations versus time and the corresponding spectrum in the acoustic range. (**b**) Observation of the disruption thresholds when using the neutral glasses and the modulated glasses for the DCD-and-dyslexia student. A 12 dBa improvement is observed.

In the first round, the student with DCD and dyslexia, wearing neutral spectacles (Figure 7a,b), detected his disruption threshold for reading at a keyboard noise of 48 dBa . Here, the noisy internal visual crowding that accompanies each word and the external keyboard computer noise add their contributions to the cross-modal areas and disrupt the person’s reading and comprehension (Figure 8). Note that the threshold is slightly lower than in experiment 1 as the angular dimension for the letters is reduced by 1.4 compared to experiment 1 with the HP screen.

In the second round, if the subject repeated the same experiment reading the text through the second pair of glasses modulated at his adapted frequency of 82 Hz (Figure 7a,b) where the extra duplicated images were erased, the disrupted threshold shifted to 60 dBa. The 12 dBa improvement corresponds to the suppression of the internal visual noise.

**Figure 9:**
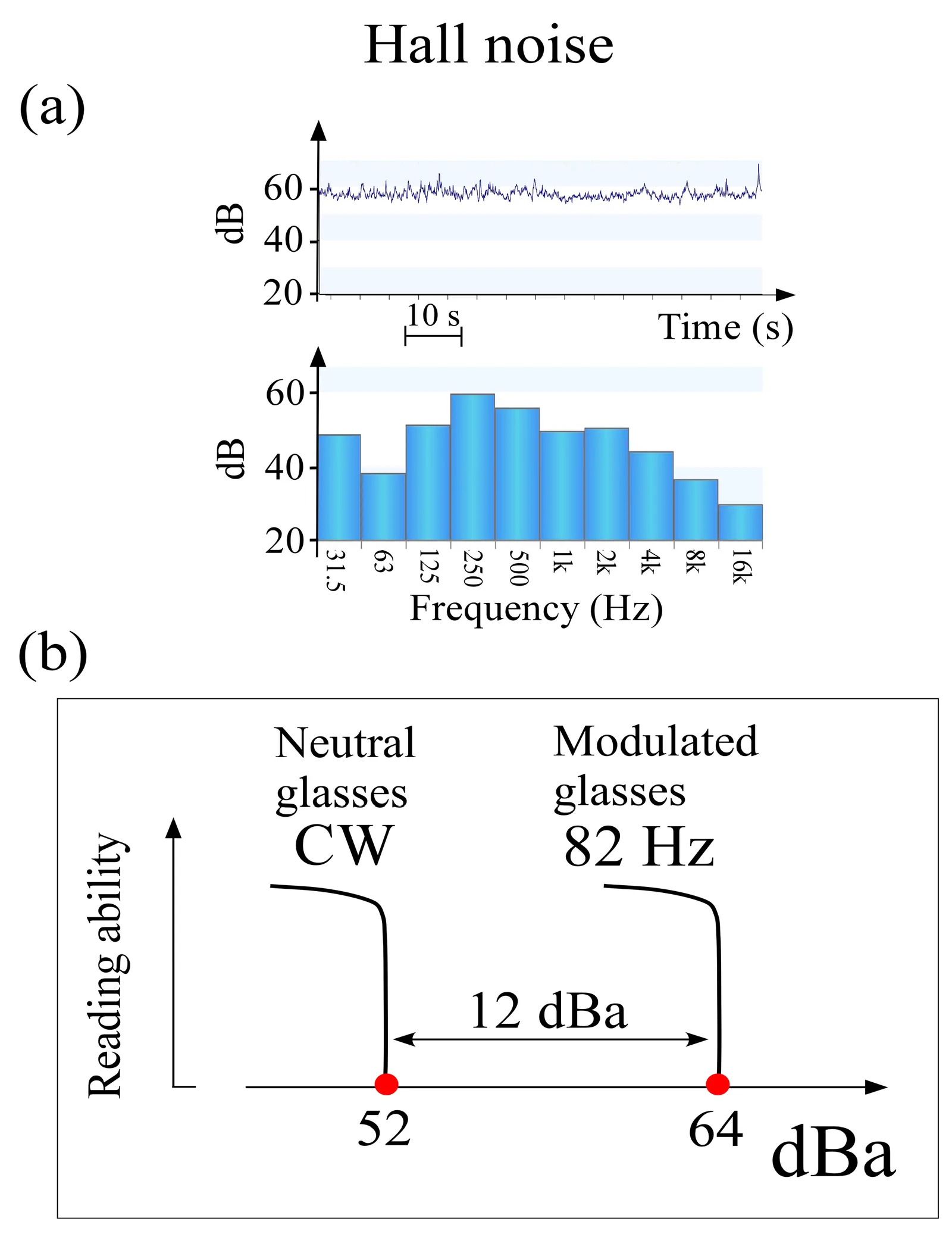
(**a**) Hall noise characteristics showing the fluctuations versus time and the corresponding spectrum in the acoustic range. (**b**) Observation of the disruption thresholds when using the neutral glasses and the modulated glasses for the DCD-and-dyslexia student. Note that the 12 dBa improvement due to the erasure of the extra duplicated afterimages is retained, but everything is shifted because the fluctuations in the intensity of this noise are smaller than those for the keyboard noise.

When the experiment was reproduced with an acoustic hall noise, the threshold with the neutral glasses occurred at 52 dBa, but this threshold shifted to 64 dBa with the modulated glasses at 82 Hz. The two thresholds slightly shifted with respect to the keyboard noise, but the gap of 12 dBa was maintained. Since the fluctuations of the noise were larger for the keyboard noise (see Figure 8) than for the hall noise (Figure 9), the perturbations were more important for this noise, and the disruption threshold was reached more rapidly. For the control, no disruption threshold was observed in the 30 to 70 dBa range in our experiment for either of the two acoustic noises.

## 4. Discussion and Conclusions

The aim of this study is to investigate and understand the role of an increased acoustic noise in the behavior of a reader with DCD and dyslexia. In particular, it is shown that a disruptive effect occurs in the 50 to 60 dBa range of acoustic noise when a cross-modal interaction between the auditory and the visual noises is involved, leading to disruption in reading. This results in a chaotic increase in the number of eye fixations that is experimentally detected by an eye tracker. In contrast, no disruption is observed in a typical reader at the same levels of acoustic noise. Cross-modal interactions are known to be ubiquitous in animal and human development and behavior and take place in the multimodal areas in the brain [18,33,34,35]. It generally involves the synergetic integration of two sensory systems. Recently, in mice, olfactory stimuli has been shown to induce excitation in the somatosensory cortex in the early postnatal period [36]. It has also been shown that different modalities of sensation are integrated even in invertebrates. The interaction between olfactory and visual learning has been isolated in Drosophila [37]. Even bumblebees perform cross-modal recognition [38]. The monomodal nuclei corresponding to vision and touch, respectively, are compared in multimodal higher centers in the brain to identify the same object in each sensory modality, as in humans, who integrate visual and haptic information quite easily [39].

Unfortunately, when the sensory systems are exposed to noises via different senses, the cross-modal interaction alters the development and behavior. Here, a reader with DCD and dyslexia is confronted with the two visual and acoustic noises. Other sensory modalities like tactile or olfactory stimuli could perhaps also interact in the cross-modal areas. Recently, it has also been shown that even 65 dB of prenatal human-generated noise directly alters the development of birds and shortens their telomeres [40]. Multisensory noise connections also seem to occur in humans with neural disorders such as autism where reduced tolerance to sound is commonly observed [41,42] with additional perturbations via vision and touch. Quiet time [43] in primary [44] and secondary [45] school classrooms can then be beneficial for learning and social relationships for individuals with such multisensory difficulties coupled with auditory difficulties. Regardless of the method, it is highly recommended to reduce the acoustic noise level itself, perhaps by using active noise reduction as suggested in Ref. [46].

To conclude, for a typical reader, external acoustic noise has no significant effect when noise is increased in the 30–70 dBa range, whereas for a reader with DCD and dyslexia, both internal and external noise cause disruption in reading when the increased acoustic noise reaches 50 to 60 dBa, depending on the type of the noise. This disruption in reading results in numerous additional fixations in eye movements quantitatively measurable with an eye tracker, which appears very effective in detecting the role of acoustic noise in reading. Fortunately, since visual noise is internal and linked to interhemispheric callosal projections, Hebbian mechanisms allow us to erase this internal visual crowding, using a pulsed-lighting regime at around 80 Hz. Then, in the presence of increasing external acoustic noise, the disruption threshold is pushed back with an improvement of 12 dBa, for both keyboard noise and hall noise. Hebbian mechanisms could be used to produce better reading capability in DCD-and-dyslexia subjects. The student in this study continues to wear the pulsed glasses every day. Although we have only compared the results of one student with DCD and dyslexia with those of a control, we hope that these results will be confirmed by other research groups using larger samples with varying degrees of DCD and dyslexia.

## 5. Patents

Patent n°FR2205481 has been filed by the authors for the foveascope.

## Data Availability

This article has no additional data.
