# Correction: Le Floch, A.; Ropars, G. Hebbian Optocontrol of Cross-Modal Disruptive Reading in Increasing Acoustic Noise in an Adult with Developmental Coordination Disorder: A Case Report. *Brain Sci.* 2024, *14*, 1208

**DOI:** 10.3390/brainsci16080870

**Published:** 2026-08-17

**Authors:** Albert Le Floch, Guy Ropars

**Affiliations:** Unité de Formation et de Recherche Sciences et Propriétés de la Matière, University of Rennes, Campus de Beaulieu, Building 7, Laboratory P938, 35042 Rennes Cedex, France; albert.lefloch@laposte.net

In the published publication [1], there was an error regarding the affiliations of both authors. With this update, the original first two affiliations have been removed, and the third affiliation has been updated to: 

“Unité de Formation et de Recherche Sciences et Propriétés de la Matière, University of Rennes, Campus de Beaulieu, Building 7, Laboratory P938, 35042 Rennes Cedex, France”.

The authors state that the scientific conclusions are unaffected. This correction was approved by the Academic Editor. The original publication has also been updated.
